# The University of Florence Technique for Robot-Assisted Kidney Transplantation: 3-Year Experience

**DOI:** 10.3389/fsurg.2020.583798

**Published:** 2020-11-11

**Authors:** Graziano Vignolini, Isabella Greco, Francesco Sessa, Luca Gemma, Alessio Pecoraro, Paolo Barzaghi, Antonio Grosso, Francesco Corti, Nicola Mormile, Marco Martiriggiano, Alessandro Berni, Niccolò Firenzuoli, Mauro Gacci, Saverio Giancane, Arcangelo Sebastianelli, Vincenzo Li Marzi, Sergio Serni, Riccardo Campi

**Affiliations:** ^1^Unit of Urological Robotic Surgery and Renal Transplantation, Careggi Hospital, University of Florence, Florence, Italy; ^2^Department of Experimental and Clinical Medicine, University of Florence, Florence, Italy

**Keywords:** deceased-donor, kidney transplantation, living-donor, minimally-invasive surgery, robotics

## Abstract

**Objective:** To report the University of Florence technique for robot-assisted kidney transplantation (RAKT) from living donor (LD) and deceased donor (DD), highlighting the evolution of surgical indications and technical nuances in light of a single surgeon's learning curve.

**Materials and Methods:** A dedicated program for RAKT from LDs was developed at our Institution in 2017 and implemented later with a specific framework for DDs. All RAKTs were performed by a single highly experienced surgeon. Data from patients undergoing RAKT between January 2017 and December 2019 were prospectively collected in a dedicated web-based data platform. In this report we provide a comprehensive step-by-step overview of our technique for RAKT, focusing on the potential differences in peri-operative and mid-term functional outcomes between LDs vs. DDs.

**Results:** Overall, 160 KTs were performed in our center during the study period. Of these, 39 (24%) were performed with a robot-assisted laparoscopic technique, both from LDs (*n* = 18/39 [46%]) and from DDs (*n* = 21/39 [54%]). Eleven (11/39 [18%]), 13(13/39 [26%]), and 15 (15/39 [30%]) RAKTs were performed in 2017, 2018, and 2019, respectively, highlighting an increasing adoption of robotics for KT over time at our Institution. Median time for arterial (19 min for LD and 18 min for DD groups), venous (21 min for LD, 20 min for DD) and uretero–vesical (18 min for LD and 15 for DD) anastomosis were comparable between the two groups (all *p* > 0.05), as the median rewarming time (59 min vs. 56 min, *p* = 0.4). The rate of postoperative surgical complications according to Clavien–Dindo classification did not differ between the two study groups, except for Clavien–Dindo grade II complications (higher among patients undergoing RAKT from DDs, 76 vs. 44%, *p* = 0.042). Overall, 7/39 (18%) patients (all recipients from DDs) experienced DGF; two of them were on dialysis at last FU.

**Conclusions:** Our experience confirms the feasibility, safety, and favorable mid-term outcomes of RAKT from both LDs and DDs in appropriately selected recipients, highlighting the opportunity to tailor the technique to specific recipient- and/or graft-characteristics. Further research is needed to refine the technique for RAKT and to evaluate the benefits and harms of robotics for kidney transplantation from DDs.

## Introduction

While still being considered an experimental procedure (1), robot-assisted kidney transplantation (RAKT) from living donors (LDs) has been recently introduced at selected referral Centers worldwide, reproducing the principles of open kidney transplantation (KT) and achieving favorable perioperative outcomes with the advantages of minimally invasive surgery (2–5).

To date, most groups performing RAKT replicated the Vattikuti-Medanta technique (1). Nevertheless, specific modifications have been proposed by several teams to adapt the technique to the availability of surgical instrumentation, logistical issues, and surgeon's preference during the learning curve (4–7). Moreover, our group reported for the first time the feasibility and safety of a structured RAKT program from deceased donors (DDs) (4).

In this report we describe the University of Florence technique for RAKT from both LD and DD, highlighting the evolution of both indications and technical nuances over a 3-year period, as well as the intraoperative, perioperative and mid-term functional outcomes.

## Materials and Methods

### Patients Selection and Dataset

A dedicated program for RAKT was developed at our Institution in 2017 (4).

**Figure 1:**
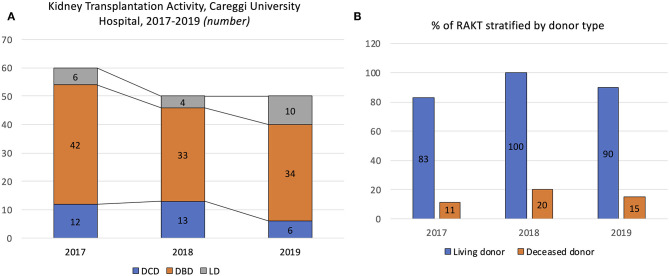
Overview of the kidney renal transplantation activity from living donor (LD) and deceased donor (DD) at our center during the study period (2017–2019). **(A)** Number of kidney transplantations performed at Careggi University Hospital in 2017, 2018, and 2019, stratified by donor type [donor after circulatory death (DCD), donor after brain death (DBD), and LD]. **(B)** Proportion of robot-assisted kidney transplantation (RAKT) performed in 2017, 2018, and 2019, stratified by donor type (deceased vs. living).

After Institutional Ethical Committee approval and obtained the patients' informed consent, data of patients undergoing RAKT from LD or DD were prospectively collected in our institutional web-based database. All consecutive patients undergoing RAKT between January 2017 and December 2019 were included in the analytic cohort (Figure 1).

At our center, the KT team includes 4 surgeons; however, all RAKTs were performed by a single surgeon (G.V.) with high experience in open KT (*n* > 100) and robotic urological surgery (*n* > 1,500). The first LD-RAKT was performed on January 25th, 2017, while the first DD-RAKT on October 16th, 2017.

LD-RAKT was always performed in a twin operating theater, specifically designed to optimize the logistics and timing of donor nephrectomy and KT.

Selection criteria for RAKT changed over time. At the beginning of our experience, strict selection criteria were followed, reserving RAKT for recipients with no previous history of surgery or KT and for grafts with no vascular abnormality (4). Later, thanks to progressive acquisition of skills and experience, we opted for RAKT also in case of more complex vascular graft anatomy requiring multiple anastomoses, for previously transplanted recipients or recipients with previous abdominal surgery.

The current exclusion criteria for RAKT at our center are therefore: (a) age <18 years; (b) absolute contraindication for robotic surgery; c) severe atherosclerotic plaques at the level of iliac vessels and (for DD-RAKT), and (d) lack of all key logistical requirements including availability of the operating theater (4).

Regarding DD-RAKT, our program included both donors after brain death (DBD) and donors after circulatory death (DCD). However, since 2019, RAKT has been prioritized for DBD donors in light of a potentially higher risk of adverse peri-operative outcomes in the DCD donor population (4).

Preoperative evaluation of donors, postoperative management, and follow-up after RAKT were performed according to the principle of the latest EAU Guidelines on KT (8).

All recipients underwent CT-angiogram to assess the vascular anatomy, potential atherosclerotic plaques and anomalies of iliac vessels before RAKT (9).

Chronic Kidney Disease Epidemiology Collaboration formula was used to calculate estimated glomerular filtration rate (eGFR) in patients aged <70 years (10), while the Berlin Initiative Study formula was used for patients aged ≥70 years (11). Patient comorbidities were recorded using Charlson Comorbidity index (CCI) (12).

Warm ischemia time was defined as the time from the circulatory arrest of the organ procured to the begging of cold storage, while cold ischemia time as the time of cold storage, until its interruption before graft introduction into the recipient.

Surgical postoperative complications were classified according to the modified Clavien–Dindo system (13).

Delayed graft function (DGF) and primary non-function (PNF) were defined as the need of dialysis in the first postoperative week and the need for dialysis after RAKT with ultrasonography confirming adequate perfusion of the graft, respectively.

### Decision-Making Regarding the Technical and Logistical Feasibility of RAKT From Deceased Donors

In the context of DDs, the following key phases were crucial to evaluate the technical and logistical feasibility of RAKT:

- *Phase 1 (team*): after the Regional Allocation Center for Organs and Tissues provided the alert of a potential donation, the feasibility of RAKT depended on the availability of the dedicated surgical team.
- *Phase 2 (recipient):* in the meantime, the recipient was admitted to the Nephrology Unit and a careful preoperative evaluation, including anesthesiologic work-up and CT scan, was performed to ensure that all patients' selection criteria are respected.
- *Phase 3 (robotic operating room):* the feasibility of RAKT also required that the robotic operating room was available. Of note, our robotic OR is available for RAKT from DDs with no restrictions during the night-time and in the week-ends.
- *Phase 4 (cold ischemia):* RAKT should be started within 16 h from the beginning of cold storage, keeping the overall cold ischemia time >24 h.
- *Phase 5 (bench evaluation of the graft vascular anatomy):* RAKT was performed only after careful evaluation of the graft at bench surgery, provided that no highly complex graft vascular anatomy (i.e., >2 arteries and/or >2 veins and/or abnormalities of renal vessels) was present. In this case, open KT is still preferred as multiple anastomoses might potentially increase the rewarming time during RAKT.

### Organ Procurement and Bench Surgery

For LD nephrectomy, the da Vinci Si robotic platform (Intuitive Surgical Inc., Sunnyvale, CA, USA) in a three-arm configuration was used, while for DDs we followed the European Association of Urology (EAU) Section of Transplantation (ESTU) guidelines for organ procurement (14). Organ procurement from DCD donors was performed according to established surgical principles according to our Institution protocol (15). For graft preservation, Celsior® solution was used in all cases.

During preparation of renal vessels at bench surgery, the anterior margin of the vein was reshaped by cutting away a slice of venous tissue to improve visualization of its posterior margin to facilitate the subsequent venous anastomosis.

In case of right-sided grafts from DDs, the length of the graft renal vein was increased during bench surgery through an inferior vena cava (IVC) patch.

**Figure 2:**
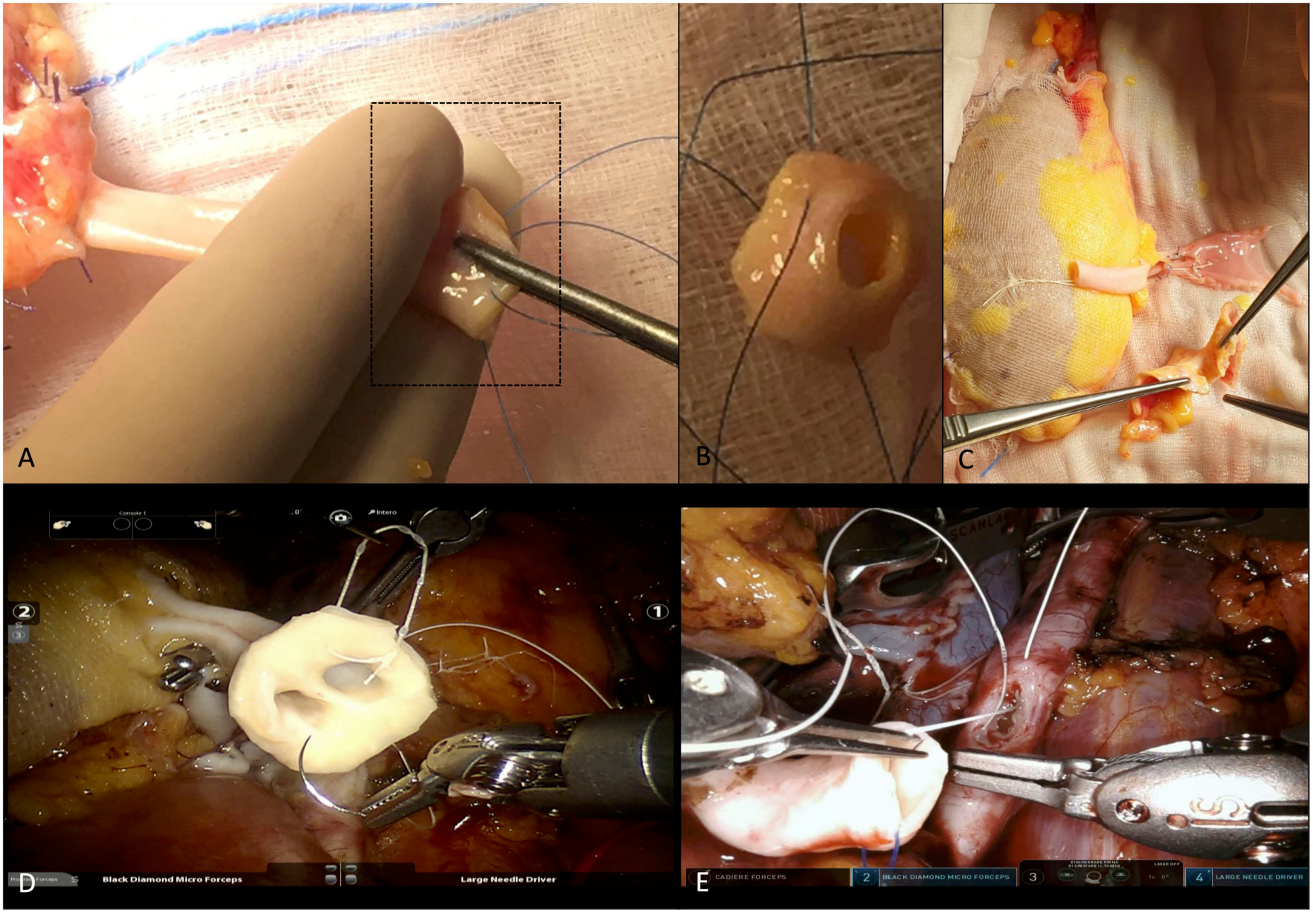
Overview of bench surgery aimed at removal of the Carrel's patch before robot-assisted kidney transplantation (RAKT) from a deceased donor (DD), and intraoperative snapshots showing two ways to perform arterial anastomosis (with and without the aortic patch). **(A–C)** The surgeon identifies the Carrel's patch and removes it before RAKT in case of aterosclerotique plaques at this level. **(D)** Intraoperative snapshot showing the performance of arterial anastomosis during a RAKT from a DD, using the Carrel's patch due to the presence of two main graft renal arteries (close to each other). **(E)** Intraoperative snapshot showing the performance of arterial anastomosis during a RAKT from a DD, without the Carrel's patch (removed during the bench surgery). In this case the surgeon opted for removal of the patch due to the presence of several plaques at the level of the graft renal artery ostium.

While at the beginning of our experience of RAKT from DDs we always used the Carrel's patch to facilitate the arterial anastomosis, from the 11th case onward (provided that no multiple arteries were present), the patch was always removed (Figures 2A–C). As the risk of atherosclerotic plaques is usually higher on the aortic patch as compared to the graft renal artery, this maneuver may improve the safety of arterial anastomosis. In addition, it may allow an easier anastomosis due to the shorter arteriotomy and a better match of caliber and shape between the graft artery and the external iliac artery.

During bench surgery, in case of multiple vessels, two techniques were used according to the graft vascular anatomy and surgeon's preference: (I) reconstruction of a single arterial vessel using the pantaloon technique to allow the performance of a single anastomosis in case of two arteries close to each other of approximately equal caliber; (II) modeling of the aortic patch to allow the performance of a single anastomosis in case of two arteries close to each other from DDs (5). In case of two arteries far from each other, two separate anastomoses were performed in an end-to-side fashion to the recipient's external iliac artery.

An ureteral stent (5 F/14 cm) was placed during bench surgery to facilitate uretero–vesical anastomosis during robotic transplantation.

The graft was finally wrapped in a gauze jacket filled with ice, with the renal artery fixed to the gauze with a landmark stich, to improve the visibility for the subsequent venous anastomosis.

### Patient Positioning and Port Placement

RAKT was performed using either the da Vinci Si or Xi robotic platform (Intuitive Surgical Inc., Sunnyvale, CA, USA) in a four-arm configuration.

Currently, we used a 0° lens and set the Trendelenburg tilt at 20°. When RAKT was performed in the left iliac fossa, an additional lateral tilt of the operative table of 5°-15° was used to better expose the left iliac vessels (in particular, the left external iliac vein).

Our surgical technique follows the principles of the Vattikuti Urology Institute-Medanta technique (1, 2) with specific technical nuances developed over time during the learning curve (4, 6).

Specifically, port placement followed the principles of robot assisted radical prostatectomy (RARP), with only one 12 mm port for the bed-side assistant and a variable triangulation toward the transplantation site at right or left iliac fossa. Pneumoperitoneum pressure was set at 8–10 mmHg and maintained constant through the use of the Airseal system. The da Vinci robot is docked between the patient's legs (Si platform®) or on the lateral side (Xi platform®), according to surgeon's preference and/or patient-specific characteristics.

For the first 17 cases, a 5 cm periumbilical incision was made on the midline to place the GelPOINT™ device (Applied Medical, Santa Ranchero, CA, USA), to allow the introduction of the graft into the peritoneal cavity.

**Figure 3:**
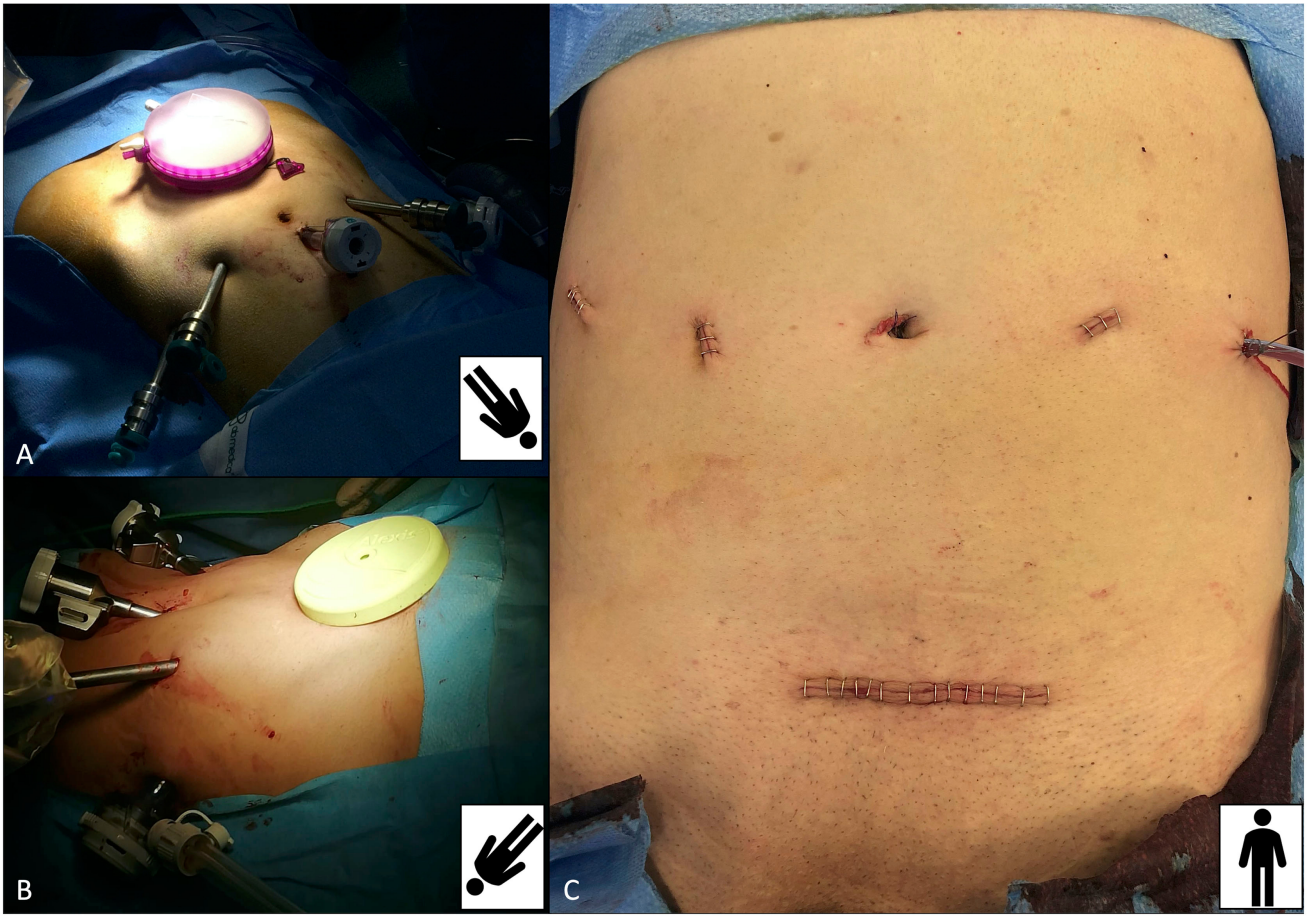
Overview of port placement for robot-assisted kidney transplantation (RAKT) and its final cosmetic result. Port placement mirrors that of robot-assisted radical prostatectomy, with the camera port placed 1–2 cm above the umbilicus, one 12-mm assistant port on the right iliac fossa and the GelPOINT device **(A)** or the Alexis retractor **(B)** placed at the level of a Pfannenstiel incision (through which the graft is inserted into the abdominal cavity). **(C)** Final cosmetic result after RAKT.

Thereafter, to reduce unnecessary costs, from the 18th case onward, the Alexis® retractor (Alexis® O Wound Retractor/Protector, Applied Medical Technology, Modesto, CA, USA) was used. Moreover, from the 18th case, a Pfannestiel incision was performed (after the complete preparation of both the iliac vessels and the bladder, re-docking the robot) to place the Alexis® device, allowing (I) better aesthetic results, (II) easier placement of the graft directly into the iliac fossa site, and (III) a direct access for the bed-side assistant to the operative field in case of intraoperative complications (Figure 3).

In case of recipients affected by Autosomal Dominant Polycystic Kidney Disease (ADPKD), not requiring previously native kidney nephrectomy, port placement resulted slightly different, to tailor the surgical strategy to the specific patient's anatomy (16).

### Dissection of Iliac Vessels, Creation of an Extraperitoneal Pouch and Preparation of the Bladder

After port placement, the peritoneum was incised at the level of the external iliac vessels, and both the external iliac artery (EIA) and external iliac vein (EIV) are skeletonized. For LD RAKT, extent of iliac vessels skeletonization was limited to the transplantation site, while for DDs (both DBD and DCD) it was extended until the bifurcation of the iliac vessels. Then, an extraperitoneal pouch was created for subsequent allocation of the graft after completion of vascular anastomoses. The Retzius space was developed, and the bladder prepared for the subsequent uretero–vesical anastomosis.

### Introduction of the Graft Into the Abdominal Cavity

After this step, the robotic instruments are temporarily removed, the robot is undocked, and the Pfannenstiel incision is performed to allow placement of the Alexis® retractor or the GelPOINT device (Figures 3A,B). This surgical step is specifically performed after the development of the Retzius space and bladder preparation to reduce the risk of any potential bladder injury.

Then, the graft is gently introduced into the abdomen through the Alexis® device, with the hilum oriented toward the site of the iliac fossa, adding 250 mL of ice into the abdominal cavity to achieve regional hypothermia.

### Venous Anastomosis

A pictorial overview of our technique for venous anastomosis is described in a previous publication (4). In some cases of right-sided grafts from DDs, the length of the graft renal vein was increased during bench surgery through an IVC cuff.

After careful evaluation of the site for venous anastomosis and placement of bull-dog clamps, a venotomy is performed on the EIV. Of note, for the first 8 cases, Potts scissors were used to perform the venotomy. Afterward, the venotomy was performed by using cold scissors, that allowed an easy modeling of the venotomy as well as a reduction in terms of costs for surgical instrumentation.

After flushing of the lumen of the external iliac vein with heparinized saline, venous anastomosis is performed in an end-to-side fashion to the external iliac vein using a 6-0 GORE-TEX suture (Gore Medical, Flagstaff, AZ, USA) on a CV-6 TTc-9 needle (5). Technique for venous anastomosis was similar for robotic transplantation using grafts from LD and DD.

### Arterial Anastomosis

An overview of the steps for arterial anastomosis is described in a previous publication (4). After modeling of the graft artery with cold scissors (to adapt its caliber to that of the EIA), a liner arteriotomy is performed and subsequently converted into a circular arteriotomy to facilitate the anastomosis. For this step, we used a robotic scalpel and aortic punch in the very first case, as previously described (1, 2), with Potts scissors from case 2 to case 8 and finally, from case 9 onward, with conventional cold scissors (4).

Moreover, at the beginning of our robotic program for DD RAKT, we performed arterial anastomosis using the Carrel's patch, provided that no significant atherosclerotic plaques were present at the level of the renal artery ostium during bench surgery.

Nowadays, the arterial anastomosis during RAKT from DDs is usually performed without the aortic patch. This approach might result in an easier anastomosis due to: (i) the shorter arteriotomy; (ii) the more anatomic anastomosis thanks to the similar caliber and structure of graft renal artery and external iliac artery, and (iii) the reduced risk of significant atherosclerotic plaques at the level of graft renal artery as compared to Carrel' s patch (4) (Figures 2D,E).

The renal artery is anastomosed in an end-to-side fashion to the external iliac artery using a 6-0 (or 5.0 in case of DDs) GORE-TEX suture on a CV-6 TTc-9 needle with two half running sutures using two different threads (4).

### Graft Reperfusion and Uretero–Vesical Anastomosis

The integrity of the venous and arterial anastomoses as well as the graft revascularization are checked through a macroscopic inspection for color and turgor. Moreover, to assess the graft and ureteral reperfusion, intraoperative duplex ultrasound and intraoperative fluorescence vascular imaging with indocyanine green (FireFly™ fluorescence imaging for Xi robotic platform) are employed, as previously reported (4, 17, 18). The graft is then placed in the previously prepared extraperitoneal pouch, partially closed with a single V-loc running suture, to avoid the potential torsion of the graft.

After checking of the ureteral reperfusion using fluorescence imaging, the uretero–vesical anastomosis is made according to a modified Lich–Gregoire technique over the pre-placed JJ stent creating an anti-refluxing mechanism, as previously described (2, 3).

### Post-operative Recovery

By Institution protocol, all recipients after KT are admitted in intensive care unit (ICU) and then in the Nephrology and Dialysis Unit for the post-operative course. A standardized institutional follow-up protocol, including serial duplex-US, was applied in all patients (9).

### Statistical Analysis

Descriptive statistics were obtained reporting medians (and interquartile ranges, IQR) for continuous variables, and frequencies and proportions for categorical variables, as appropriate.

Differences in baseline donor, graft, and recipient characteristics, as well as differences in peri-operative, and functional outcomes between the LD and DD RAKT cohorts were assessed using the Pearson's Chi-square and Mann-Whitney *U* tests, as appropriate.

Due to the limited sample size, no formal comparison of intra- or postoperative outcomes between robotic and open KT from DDs was performed.

Statistical analyses were performed using SPSS v. 24 (IBM SPSS Statistics for Mac, Armonk, NY, IBM Corp). All tests were two-sided with a significance set at *p* < 0.05.

## Results

### Overview of the RAKT Program at Careggi University Hospital During the Study Period

Overall, 160 KTs were performed in our center during the study period. Of these, 39 (24%) were performed with a robotic technique (18/39 [46%] from LDs; 21/39 [54%] from DDs) (Figure 1). Considering the whole study period (2017–2019), 11 (11/39 [18%]), 13 (13/39 [26%]), and 15 (15/39 [30%]) RAKTs were performed in 2017, 2018, and 2019, respectively, highlighting an increasing adoption of robotics for KT over time. As proof of this trend, RAKTs from DBD donors progressively increased in our minimally invasive program (10, 18, and 18% in 2017, 2018, and 2019, respectively), while in the setting of living donation, RAKT represented the preferred surgical strategy (83, 100, and 90% in 2017, 2018, and 2019, respectively).

Notably, in our series RAKT from DDs was never impeded by the unavailability of the robotic OR. Yet, during the early phase of the learning curve, two patients were not deemed eligible for RAKT despite the availability of the surgeon due to a previous KT in the right iliac fossa (open KT in the left iliac fossa was preferred in both cases).

### Characteristics of the Study Cohort

Preoperative donor, recipient and graft characteristics are shown in Table 1. Specifically, 22/39 (56%) RAKT used left-sided grafts. Double arteries were found in one graft from LD (1/18 [6%]) and in 6 (6/21 [29%] grafts from DD.

**Table 1:** Preoperative characteristics of donors, recipients, and grafts, stratified by donor type (living vs. deceased).

		**Living donors (*n* = 18)**	**Deceased donors (DBD or DCD) (*n* = 21*)***
**Donor**			
**Male gender** (*n*, %)		9 (23)	13 (33)
**Age**, years (median, IQR)		58 (48–65)	49 (39–54)
**BMI**, kg/m^2^ (median, IQR)		26 (22–29)	24 (22–28)
**Donor with hypertension** (*n*, %)		8 (44)	3 (14)
**Expanded criteria donors** (ECD) (*n*, %)		7 (39)	6 (29)
**eGFR**, mL/min/1.73 m^2^ (median, IQR)		93 (86–110)	82 (61–90)
**Graft**			
**Left kidney side** (*n*, %)		13 (72)	9 (43)
**WIT**, seconds (median, IQR)		210 (120-300)	–
**CIT**, hours (median, IQR)		1 (1–1.5)	17.5 (16–19.5)
**Biopsy of the graft** (*n*, %)		0 (0)	10 (48)
**Karpinski score** (tot = 10) (*n*, %)		–	2 (*n* = 5); 4 (*n* = 5)
**Multiple arteries grafts** (*n*, %) (*n* = 2 arteries)		1 (6) *(Separate end-to-side anastomoses to the EIA)*	6 (29) *(one single anastomosis after bench reconstruction or using the Carrel's patch)*
**Multiple veins grafts** (*n*, %) (*n* = 2 veins)		1 (6) *(Separate end-to-side anastomoses to the EIV)*	0 (0.0)
**Recipient**			
**Male gender** (*n*, %)		9 (50)	13 (62)
**Age**, years (median, IQR)		48 (39–57)	45 (36–54)
**BMI**, kg/m^2^ (median, IQR)		23.5 (21.1–26.7)	23.3 (21.0–24.9)
**Charlson comorbidity index** (median, IQR)		2 (2–3)	2 (2–3)
**ASA® score** (median, IQR)		2 (2–3)	2 (2–3)
**Nephropathy** (*n*, %)	Post infectious GN	1 (6)	2 (10)
	IgA nephropathy	3 (17)	2 (9)
	FSGS	2 (11)	0 (0)
	MGN	1 (6)	1 (5)
	MPGN	1 (6)	0 (0)
	Lupus nephritis	0 (0)	3 (14)
	Schönlein–Henoch purpura	1 (6)	1 (5)
	ADPKD	2 (11)	4 (19.0)
	DM nephropathy	0 (0)	1 (4.8)
	Others	7 (38.9)	7 (33.3)
**Native nephrectomy** (*n*, %)		0 (0.0)	1 (4.8)
**Major previous abdominal surgery** (*n*, %)		2 (11.1)	2 (9.5)
**Previous transplantation** (*n*, %)		0 (0.0)	1 (4.8)
**Recipient in treatment with antiplatelets or anticoagulants** (*n*, %)	Antiplatelet	0 (0.0)	2 (9.5)
	Anticoagulant	1 (5.6)	1 (4.8)
**Pre-emptive recipients** (*n*, %)		9 (50.0)	7 (33.3)
**Duration of dialysis** (*n* = 23) (months) (median, IQR)		22 (9–48)	20 (12–46)
**Type of Dialysis** (*n* = 23) (*n*, %)	Haemodialysis	7 (77.8)	11 (78.6)
	Peritoneal dialysis	2 (22.2)	3 (21.4)
**Preoperative Hb**, g/dL (median, IQR)		12 (10–12)	11 (10–12)
**Preoperative eGFR**, mL/min/1.73 m^2^ (median, IQR)		9 (5–11)	9 (7–12)

*ECD, extended criteria donors (donor's age >60 years or >50 years with two of the following: history of high blood pressure, creatinine ≥ 1.5 mg/dL, or death resulting from a stroke, according to The Organ Procurement and Transplantation Network (OPTN) criteria); eGFR, estimated glomerular filtration rate (CDK-EPI formula); CIT, cold ischemia time; WIT, warm ischemia time; ADPKD, autosomal dominant polycystic kidney disease; ASA, American Society of Anesthesiologists Classification; BMI, body mass index; DM, diabetes mellitus; FSGS, focal segmental glomerulosclerosis; GN, glomerulonephritis; MGN, membranous glomerulonephritis; MPGN, membranoproliferative glomerulonephritis*.

*Italic values describe detail on the graft characteristics*.

Median (IQR) cold ischemia time (CIT) was 1 h (1–1.5) for LDs while 17.5 h (16–19.5) for DDs. Median (IQR) BMI was 26 (21–27) and 24 (21–25) in the LD and DD groups, respectively.

Overall, in the setting of LD RAKT, the graft was introduced through a periumbilical incision in 33.3% while through a Pfannestiel incision in 66.7%. These proportion were 19 and 81% in the setting of DD RAKTs (*p* = 0.3).

In all RAKTs from LD, the kidney was transplanted in the right iliac fossa, while in DD RAKTs the site was the right iliac fossa in 18 cases (85.7%) and the left iliac fossa in 3 cases (14.3%).

### Intraoperative Outcomes

Overall, median (IQR) console time was 190 (160–240) and 180 (160–220) min for LD and DD RAKTs, respectively (*p* = 0.3). Similarly, median (IQR) rewarming time was not significantly different among the two cohorts [59 min (49–72) for LD and 56 min (50–64); *p* = 0.4].

**Figure 4:**
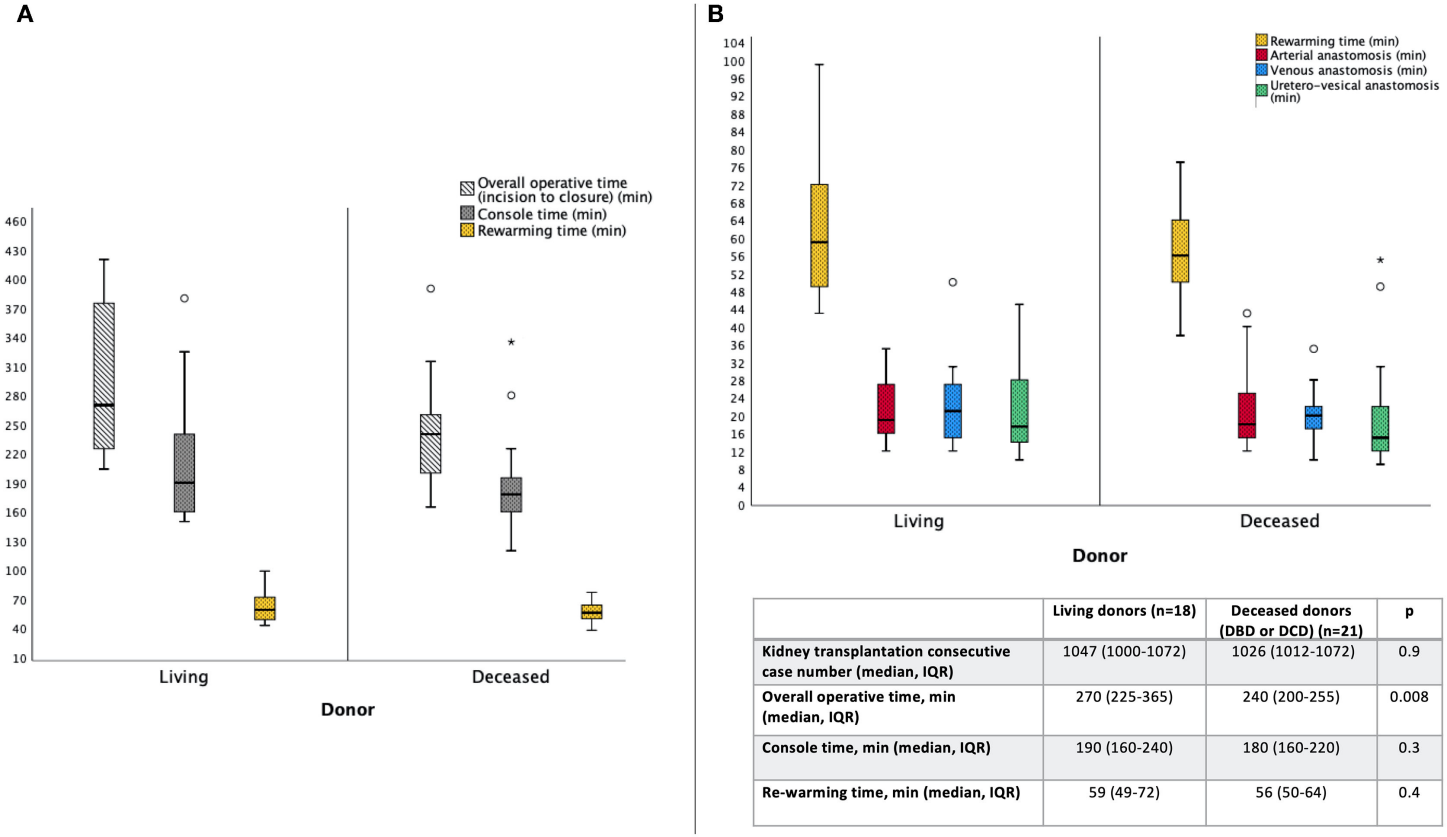
Overview of the median (IQR, range) overall operative time, console time and rewarming time **(A)**, as well as the times to complete arterial, venous and uretero–vesical anastomoses **(B)**, during robot-assisted kidney transplantation (RAKT), stratified by donor type (living vs. deceased).

Median (IQR) overall operative time was 270 min (225–365) and 240 min (200–255) in the two cohorts, respectively (*p* = 0.008) (Figure 4A).

Of note, as shown in Figure 4B, median time of arterial (19 min for LD and 18 min for DD groups), venous (21 min for LD, 20 min for DD) and uretero–vesical (18 min for LD and 15 for DD) anastomosis were comparable between the two groups (all *p* > 0.05).

**Figure 5:**
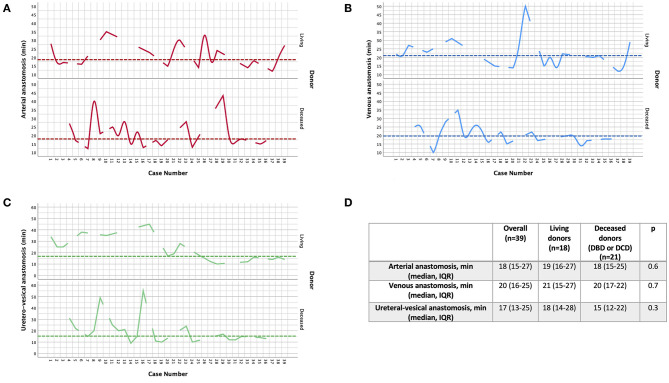
Overview of the variability in the time required to complete arterial **(A)**, venous **(B)** and uretero–vesical anastomoses **(C)** during robot-assisted kidney transplantation (RAKT), stratified by donor type (living vs. deceased) **(D)**. The x-axis shows the consecutive number of cases (from January 2017 to December 2019), while the y-axis the time (minutes) required for each anastomosis. The dotted lines represent the median values.

Moreover, as depicted in Figure 5, the times required to complete arterial, venous, and uretero–vesical anastomoses progressively decreased over time throughout the learning curve, except for highly selected cases (outlined by the spikes in the graphs).

In one case from a DD (1/39 [2.5%]), an intraoperative bleeding requiring the positioning of an extra 5 mm trocar was recorded, without need for intraoperative transfusions or conversion to open surgery.

### Postoperative and Functional Outcomes

Table 2 shows the peri-operative and functional outcomes at a median follow-up of 16 months (IQR 7–22).

**Table 2:** Peri-operative outcomes and functional outcomes after robot-assisted kidney transplantation (RAKT), stratified by donor type (living vs. deceased).

		**Donor type**			***p***
		**Overall (*n* = 39)**	**Living donors (*n* = 18)**	**Deceased donors (DBD or DCD) (*n* = 21)**	
**Peri-operative outcomes**					
**LOH in intensive care unit**, days (median, IQR)		2 (1–3)	2 (1–3)	2 (2–3)	0.3
**Overall LOH**, days (median, IQR)		14 (10–20)	14 (11–19)	14 (10–22)	0.8
**Postoperative graft biopsy** (*n*, %)		6 (15.4)	3 (16.7)	3 (14.3)	0.8
**Rejection** (*n*, %)		1 (2.6)	1 (5.6)	0 (0.0)	0.2
**FU**, months (median, IQR)		16 (7-22)	9 (7-21)	18 (8-22)	0.4
**Graft nephrectomy** (*n*, %)		1 (2.6)	0 (0.0)	1 (4.8)	0.3
**Surgical complications** *(Clavien Dindo classification)* (*n*, %)	Grade 1	15 (38.4)	10 (55.6)	5 (23.8)	0.08
	Grade 2	24 (61.5) *(n = 3 [7.7%] transfusion)*	8 (44.4) *(n = 1 [5.6%] transfusion)*	16 (76.2) *(n = 2 [9.5%] transfusions)*	**0.042** (0.6)
	Grade 3a	4 (10.3)	2 (11.1) (n = 1 ureteral stenosis requiring percutaneous placement of nephrostomy tube and JJ stent^*^; n = 1 percutaneous drainage of symptomatic lymphocele)	2 (9.5) *(n = 1 TRAS requiring PTA + stenting; n = 1 ureteral stenosis requiring percutaneous placement of nephrostomy tube and JJ stent)*	0.8
	Grade 3b	1 (2.6)	0 (0.0)	1 (4.8) *(n = 1 graft nephrectomy for arterial thrombosis [DCD donor])*	0.3
	Grade 4–5	0 (0.0)	0 (0.0)	0 (0.0)	-
**Postoperative Hb**, g/dL (median, IQR)	POD 1	11 (10–12)	11 (10–11)	10 (9–12)	0.50
	At hospital discharge	10 (9–10)	10 (9–10)	9 (9–10)	0.10
**Functional outcomes**					
**eGFR**, mL/min/1.73 m^2^ (median, IQR)	POD 1	11 (8–17)	16 (12–18)	8 (7–11)	**<0.001**
	POD 3	33 (9–43)	43 (33–48)	10 (8–35)	**<0.001**
	POD 7	41 (14–58)	54 (44–60)	16 (9–38)	**<0.001**
	At hospital discharge	51 (34–59)	54 (47–66)	39 (29–56)	0.059
	At last follow-up	55 (43–73)	51 (42–71)	57 (45–76)	0.6
**DGF** (*n*, %)		7 (17.9)	0 (0.0)	7 (33.3)	**0.007**
**Dialysis at FU** (*n*, %)		2 (5.1)	0 (0.0)	2 (9.5) *(n = 1 graft nephrectomy; n = 1 PNF [both DCD donors])*	0.1

*DGF, delayed graft function (defined as need of dialysis in the first week after transplantation); eGFR, estimated glomerular filtration rate (CDK-EPI formula); FU, follow-up; LOH length of hospitalization; POD, postoperative day*.

* *In this case, the graft showed a uretero-pelvic junction obstruction (UPJO), not requiring intervention on bench surgery; however, during the postoperative period, the patient developed hydronephrosis and required percutaneous placement of a nephrostomy tube with antegrade placement of a double J stent; the stent was removed after 3 months with no further medical or surgical complications. Bold values highlight the statistically significant results. Italic values describe detail on surgical complications*.

Specifically, the rate of postoperative surgical complications according to Clavien–Dindo classification (CDC) did not differ between the two study groups, except for the rate of Clavien–Dindo grade II complications, which were higher among patients undergoing RAKT from DDs (76 vs. 44%, *p* = 0.042). Nevertheless, of Clavien–Dindo grade II complications, only one (1/8, [12.5%]) in the LD group and two (2/16 [12.5%]) in the DD group were represented by peri-operative blood transfusions.

In our cohort, 5/39 (13%) major surgical complications were recorded. Of these, 4 (2 in the LD and 2 in the DD group) were Clavien–Dindo grade IIIa and 1 IIIb Clavien–Dindo grade complication (in the DD group). In particular, 2 recipients required percutaneous placement of a nephrostomy tube with antegrade placement of a JJ stent; in one recipient a symptomatic lymphocele was treated with percutaneous drainage and in one patient a transplant arterial stenosis (TRAS) required percutaneous transluminal angioplasty (PTA) and stenting. One patient in the RAKT DD group required graft nephrectomy due to an arterial thrombosis (in this case, the donor was a DCD) (Table 2).

Regarding functional outcomes, 7/39 (18%) patients (all recipients from DD) experienced DGF. Two of them were on dialysis at last FU (one patient after graft nephrectomy and one patient experiencing primary non-function, both cases from DCDs).

An increasing trend of eGFR was progressively recorded from the first postoperative day to the last FU (Table 2). In particular, at the last follow up visit, median (IQR) eGFR was 51 (42–71) and 57 (45–76) mL/min/1.73 m^2^ in the LD and DD groups, respectively (*p* = 0.6).

## Discussion

Although RAKT is still being performed only at selected referral Centers worldwide, current evidence confirms is feasibility, safety and reproducibility (1, 2, 19, 20). In fact, RAKT with regional hypothermia in the setting of “elective” living donation has been shown to achieve favorable short- and mid-term peri-operative and functional outcomes (mirroring the principles of open KT) while providing the key advantages of minimally-invasive surgery (2, 3, 6, 8, 19–23). Moreover, recent studies demonstrated that RAKT can be safely performed by experienced robotic surgeons even in case of grafts with vascular anomalies (5) or complex recipients such as those with ADPKD (16) or morbid obesity (7, 24, 25).

Nevertheless, the introduction of RAKT might be challenging from surgical, economical and logistical perspectives; such challenges may indeed hinder its widespread adoption in Transplant Centers. In particular, the availability and organization of a robotic operating room (OR) in condition of “emergency,” coupled with a compelling need to check the donor-, recipient-, and graft-related selection criteria for RAKT in a relatively short timeframe (as well as to keep CIT to a minimum) may represent barriers for implementation of RAKT programs from DDs.

As such, there is still lack of evidence on the outcomes of RAKT from DDs, though extending the number of minimally invasive KTs in both Urologic and Transplant Centers is becoming an increasingly relevant need.

In this scenario, taking advantage of previous pioneering studies (1, 2), in 2017 we developed a structured, dedicated RAKT program at our Institution, aiming to integrate robotics into routine KT practice from both LDs and DDs (4).

In this report we reported an updated step-by-step overview of the University of Florence technique for RAKT and its mid-term outcomes.

A key finding from our experience is that RAKT was technically and logistically feasible in a wide range of several clinical settings (LDs, DDs, grafts with multiple vessels, grafts with severe calcifications of the renal artery ostium, obese patients, etc.). Of note, all RAKTs in our series were performed by a single surgeon; the analysis of his learning curve allows to transparently evaluate the steps required to translate the knowledge and expertise in robotic urologic surgery to KT. As a proof of concept, no recipients required conversion to open surgery, and RAKT was successfully performed even at challenging times (i.e., during the weekend or at night-time) in case of DDs. While the technique for RAKT did not significantly differ between LDs and DDs, except for specific nuances such as the management of the Carell's patch (Figure 2), from a logistical perspective RAKT from DDs is far more demanding for the KT team. In this regard, to ensure a safe pathway for DD-RAKT, a well-structured decision-making process involving a multidisciplinary team of experts (urologists, nephrologists, operating room staff, etc.) must be provided (4).

From a surgical standpoint, the codified University of Florence technique for RAKT represents the evolution of the Vattikuti-Medanta technique with specific technical modifications introduced by our surgical team over time. Such modifications were introduced with the aim to simplify the technique, improving its reproducibility and allowing a larger number of (robotic) transplant surgeons to safely approach the procedure. In brief, the main technical nuances introduced by our group included: (a) the performance of a Pfannestiel incision for graft insertion into the abdominal cavity, to have direct access to the iliac fossa while improving the cosmetic result; (b) the systematic use of two single threads for performance of the arterial anastomosis, to ensure a watertight anastomosis avoiding the risk of reducing its caliber; (c) the removal of the Carrel's patch during bench surgery in cases of DD-RAKTs; (d) the performance of the venous anastomosis without the IVC patch in few cases of right-sided grafts from DDs; (e) the use of intraoperative fluorescence vascular imaging to assess the graft and ureteral reperfusion. Notably, intraoperative fluorescence vascular imaging may allow surgeons to tailor the length of the ureter according to the degree of its vascularization before uretero–vesical anastomosis, aiming to reduce the risk of subsequent stenosis of the uretero–vesical anastomosis. While promising, this technique requires further evaluation (17).

Our experience also suggests that the financial burden of RAKT might be at least partly reduced by refining the cost-effectiveness of surgical instrumentation (i.e., using the Alexis® retractor as compared to the GelPOINT™ device; avoiding the use of the aortic punch and using the conventional robotic scissors instead of the Potts scissors for the arteriotomy; using just one needle holder, etc.). Of note, such revision of the surgical instrumentation did not influence the intraoperative or postoperative outcomes, in part being counterbalanced by increasing surgical dexterity and confidence during the learning curve.

Our experience confirms that RAKT achieved favorable intraoperative, peri-operative, and functional outcomes at both a short- and mid-term follow-up (Table 2), as previously reported (6, 26). In particular, the times required for arterial, venous and uretero–vesical anastomosis were at least non-inferior to those reported in the literature for open KT (7, 14), even considering the influence of our team's learning curve (6). Moreover, while the overall surgical morbidity of RAKT in our series was acceptable and comparable to the open KT series (14), most adverse events (including major complications and DGFs) occurred in the DD-RAKT cohort, suggesting a higher peri-operative risk for these patients (Table 2).

Despite being one of the few prospective experiences on RAKT worldwide, our study is not devoid of limitations. First, it is a preliminary experience with a relatively small sample size and only a mid-term follow-up. Second, we carefully selected the recipients for RAKT, especially at the beginning of the learning curve. In addition, while the current exclusion criteria are less stringent, DD-RAKT might have not been performed at our center due to logistical challenges, introducing a selection bias. Third, in light of the study design and of the limited sample size, we could not formally compare the outcomes of RAKT and the current gold standard (open KT) in both the LD and DD setting, nor evaluate the impact of the surgeon's learning curve on peri-operative and functional outcomes. Finally, in light of the demanding logistical challenges and financial costs of RAKT (especially from DDs), the generalizability of our findings may be limited to selected referral centers.

Acknowledging these limitations, our experience provides additional evidence to further explore the potential added value of robotics for KT in both the LD and DD settings.

Further research is needed to: (a) evaluate whether RAKT provides incremental benefits for surgeons in both “standard” and “complex” cases, and whether these benefits translate into measurable improvements of the surgeons' learning curve and of patient outcomes; (b) assess the cost-effectiveness of RAKT from LDs and DDs using appropriate, standardized metrics; (c) to explore the impact of rewarming time on long-term functional outcomes; and (d) to refine the technique for RAKT by means of extended-reality platforms and/or immersive technologies such as 3D printing (27).

## Data Availability Statement

The data analyzed in this study is subject to the following licenses/restrictions: Requests to access these datasets should be directed to riccardo.campi@unifi.it.

## Ethics Statement

The studies involving human participants were reviewed and approved by Comitato Etico Area Vasta Centro—AOU Careggi. The patients/participants provided their written informed consent to participate in this study.

## Author Contributions

RC, FS, GV, and SS: study design. NM, PB, FC, AG, AB, AP, LG, NF, and MM: data collection. RC: statistical analysis. GV, RC, IG, and FS: manuscript writing. MG, AS, VL, and SG: critical revision of the manuscript. SS: supervision. All authors contributed to the article and approved the submitted version.

## Conflict of Interest

The authors declare that the research was conducted in the absence of any commercial or financial relationships that could be construed as a potential conflict of interest.
